# A New Helical Crossed-Fibre Structure of β-Keratin in Flight Feathers and Its Biomechanical Implications

**DOI:** 10.1371/journal.pone.0065849

**Published:** 2013-06-10

**Authors:** Theagarten Lingham-Soliar, Nelisha Murugan

**Affiliations:** 1 Life Sciences, University of KwaZulu-Natal, Durban, KwaZulu-Natal, South Africa; 2 Electron Microscopy Unit, University of KwaZulu-Natal, Durban, KwaZulu-Natal, South Africa; University of Sussex, United Kingdom

## Abstract

The feather aerofoil is unequalled in nature. It is comprised of a central rachis, serial paired branches or barbs, from which arise further branches, the barbules. Barbs and barbules arise from the significantly thinner lateral walls (the epicortex) of the rachis and barbs respectively, as opposed to the thicker dorsal and ventral walls (the cortex). We hypothesized a microstructural design of the epicortex that would resist the vertical or shearing stresses. The microstructures of the cortex and epicortex of the rachis and barbs were investigated in several bird species by microbe-assisted selective disassembly and conventional methods via scanning electron microscopy. We report, preeminent of the finds, a novel system of crossed fibres (ranging from ∼100–800 nm in diameter), oppositely oriented in alternate layers of the epicortex in the rachis and barbs. It represents the first cross-fibre microstructure, not only for the feather but in keratin per se. The cortex of the barbs is comprised of syncitial barbule cells, definitive structural units shown in the rachidial cortex in a related study. The structural connection between the cortex of the rachis and barbs appears uninterrupted. A new model on feather microstructure incorporating the findings here and in the related study is presented. The helical fibre system found in the integument of a diverse range of invertebrates and vertebrates has been implicated in profound functional strategies, perhaps none more so potentially than in the aerofoil microstructure of the feather here, which is central to one of the marvels of nature, bird flight.

## Introduction

Understanding biological materials such as β-keratin is exacerbated by the hierarchical organization that is inherent to the design i.e. their complexity evolved over millions of years and involves multifunctional materials. Hence, composition, structure and function are hard to separate whereas in synthetic systems there is a disciplinary separation [1]. Obtaining structural data in feather keratin is particularly hampered by the tight bond between the polymeric filaments of β-keratin and the amorphous polymer matrix. A previous contradictory claim about feather structure is that β-keratogenic tissue of the rachis and barbs was fully characterized ultrastructurally by histodifferentiation [2], [3], i.e. the bulk of the rachis, calamus, and barb rami are comprised of typical, tile-like, stratified squamous epithelial tissues. A subsequent study [4] showed that the major fibre type of the rachis was rather, a cylindrical form, elongated proximo-distally i.e., a syncitial barbule cell ∼6–8 µm in diameter (previously only known in the free barbules and down feathers). Given the claim that barbs were also comprised of tile-like cells [2], [3] it was important to add to the research agenda below an investigation on whether or not the syncitial barbule cells also typified the structure of the barb cortex, despite its much smaller size compared to that of the rachis. Besides morphology and function there are also potential evolutionary implications (Discussion).

Feathers are the most complex integumentary structures known. The flight feathers of birds must possess among their most important characteristics two almost paradoxical qualities–they must first, be composed of extremely light materials and second, be strong enough to withstand the immense aerodynamic loads experienced during flight. Despite this, most anatomical investigations portray the feather microstructure as relatively simple, particularly with respect to its microfibril organization –of fibres and fibrils that range in size from about 20 A in diameter to bundles of about 200 nm that are predominantly longitudinally oriented [5], a view that had not changed in decades until recently ( [4] and references therein). It is clear that modes of investigation of for instance feather developmental structure (e.g. by histodifferentiation [2]) on the one hand and feather functional structure on the other [4], are not mutually inclusive.

Birds groom and repair their feathers regularly because it is vital to maintaining efficient aerodynamic surfaces. The process of nibbling or “zipping” the barbs together by means of minute hook-like barbules at their ends involves considerable lateral flexibility (proximo-distal movement of the barb in relation to the rachis’ long axis). The thin cross-sectional structure of the lateral walls of the barb and much thicker dorsal and ventral walls relative to the lateral is considered significant in allowing lateral flexibility and maintaining vertical stability. These contrasting qualities are based on the gross anatomical characteristics of the barb. Yet, despite the important aerodynamic functions of the feather vane, investigations with respect to its microfibrillar construction from a functional perspective have been neglected. The present hypothesis is that the clear lateral flexibility of the rachis and barbs suggests an anisotropic microstructural fibre design of the lateral walls, to which barbs and barbules are attached, which would respond to shear and torsional stresses (vertical loads). It is the basis for the main microstructural investigations of the feather here.

Our understanding of the functional biology of the feather and its role in the evolution of bird flight implicitly requires an integration of data on its fibrillar microstructure and the more discernable gross anatomy. We use the natural keratinophylic microbial fauna of the feather as a tool to investigating its fibre structure and organization by selective biodegradation of the filament-matrix texture of β-keratin [4]. Reasons for the selective biodegradation of feather keratin are speculative. It might e.g. have a chemical explanation involving differences in the sulphur fractions in the proteins present in the amorphous matrix and in the β-keratin microfibrillar component ( [4] and references therein). However, with increasing attention to the study of keratins with respect to their genetics [6], [7], molecular structure [8], [9] and chemistry [10], finite answers to issues raised by e.g. our selective biodegradation experiments on β-keratin may just be a matter of time.

## Results

**Figure 1:**
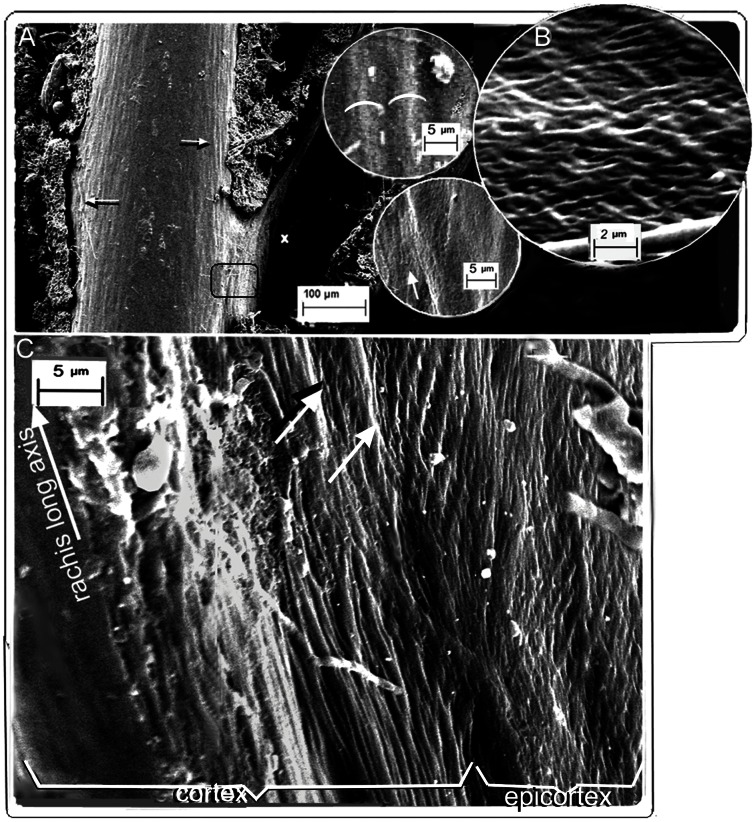
SEM of fungal matrix degraded feathers. *Gallus gallus*. (A) Cortex, dorsal surface, slight fungal degradation exposing surfaces of syncitial barbules. Insets show detail of degraded surface of rachidial cortex and partially exposed syncitial barbules (identified by characteristic thickness ∼5.0 µm; white semicircles). Upper and lower insets are details marked by right and left arrows respectively in (A). (B) Lateral tangential section of the epicortex of the rachis just below the barb base (marked x in (A)) showing cross-fibre structure. (C) Boundary between cortex and epicortex (approx. area in black rectangle in (A); arrows show the boundary where the cortex overlaps the epicortex, with a degraded area between arrows showing underlying epicortex with cross-fibres). Fungi can be seen at bottom of image.

**Figure 2 pone-0065849-g002:**
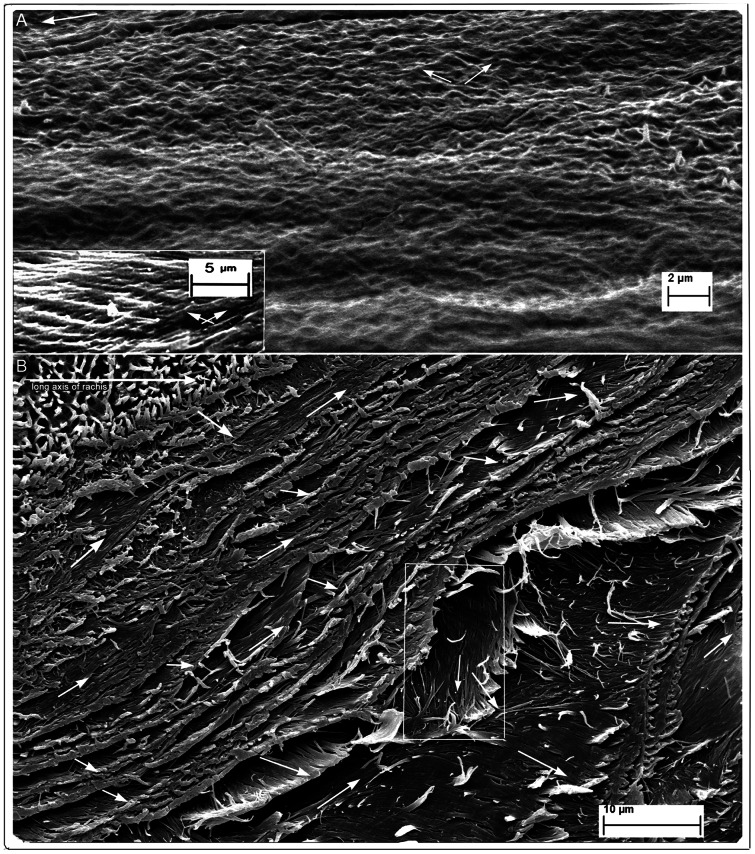
SEM of feathers of *Gallus gallus* and *Falco peregrinus*. (A) *Gallus gallus*. Fungal degraded. Rachis epicortex between successive barbs. Patches of cuticular microvilli (middle, right; also see Fig. 2B) indicates section is at the surface. Successive layers of cross-fibres form a meshwork.The rippling effect represents fibre loss of tension (possibly from wear) nearest surface (see text; left to right = long axis of rachis). Lower inset rectangle (detail from Fig. 1C) shows two geodesic cross-fibre layers from a section in which fibres are under tension (straight). (B) *Falco peregrinus*. Native (non-biodegraded). Rachis epicortex adjacent to barb. Transverse section of entire depth of epicortex, cut at acute angle, shows numerous fibre layers as they naturally occur with matrix intact. Section shows approximately 16 layers, each comprised of a two-ply of oppositely oriented fibres (see rectangle). Top left shows epicortex surface with villus cuticle intact while bottom right tapers sharply to near tangential plane to union with barb (also see details in Fig. S4A, B and S5).

**Figure 3 pone-0065849-g003:**
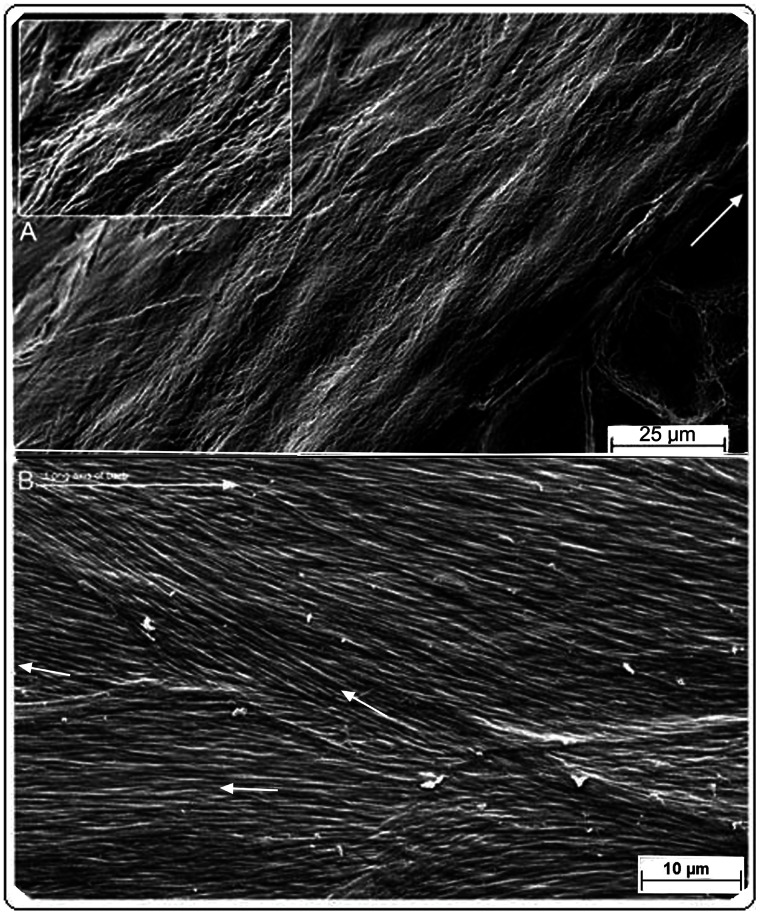
SEM of feather rachis and barb epicortex. (A) Rachis epicortex. Native (non-biodegraded). *Otus leucotis.* Tangential section (resin embedded and etched) in the left lateral wall of the rachis between adjacent barbs and very close to the medulloid pith layer below. Relief impressions of the medulloid pith cells can be seen on the overlying epicortical layer (dissected cells can be seen below). Inset, detail showing how epicortical fibre angles may change under pressure (also see Figure S1E). (B) Barb epicortex. Native (non-biodegraded). *Bubo africanus*. Tangential section (parallel to surface) showing at least three fibre layers with different fibre orientations (small arrows; top, long arrow = rachis long axis).

**Figure 4 pone-0065849-g004:**
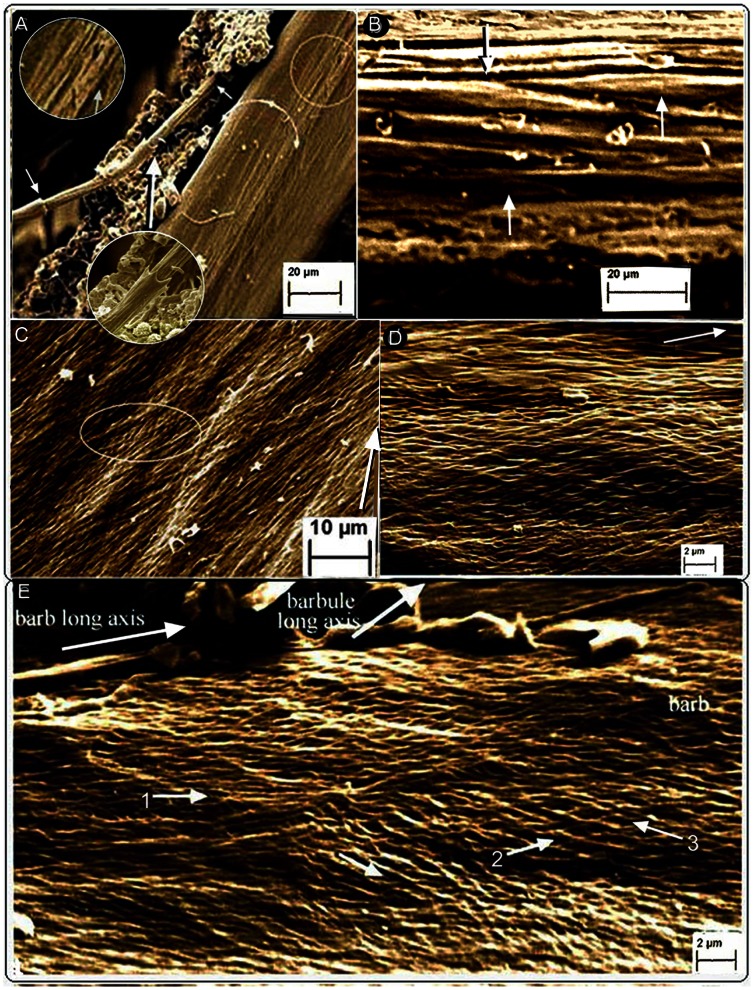
SEM of barb cortical and epicortical microstructure. Fungal selective matrix disassembly of *Gallus gallus* (A, D, E). (A) Dorso-lateral view of fungal action lifting a syncitial barbule from the left dorso-lateral surface of the barb cortex (see Fig. S1A). The arrowed hemi-circle indicates the cortex. Below it is the epicortex (right). (B) Native (non-biodegraded) feather. *Falco peregrinus.* Longitudinally sectioned barb cortex showing syncitial barbules (SI Fig.2E and inset). (C) Native (non-biodegraded) feather. *Otus leucotis*. Layers of barb epicortex fibres close to medulloid pith, closely packed with only traces of underlying layer (within oval) detectable. Impressions from the medullary cells underneath can be seen (elongated, bright areas). (D, E) Epicortex. (D) Cross-fibres of epicortex below and around barbules. (E) Several alternating layers of oppositely oriented fibres just below barbules (some outermost fibres are parallel with the barb long axis, arrow 1).

**Figure 5:**
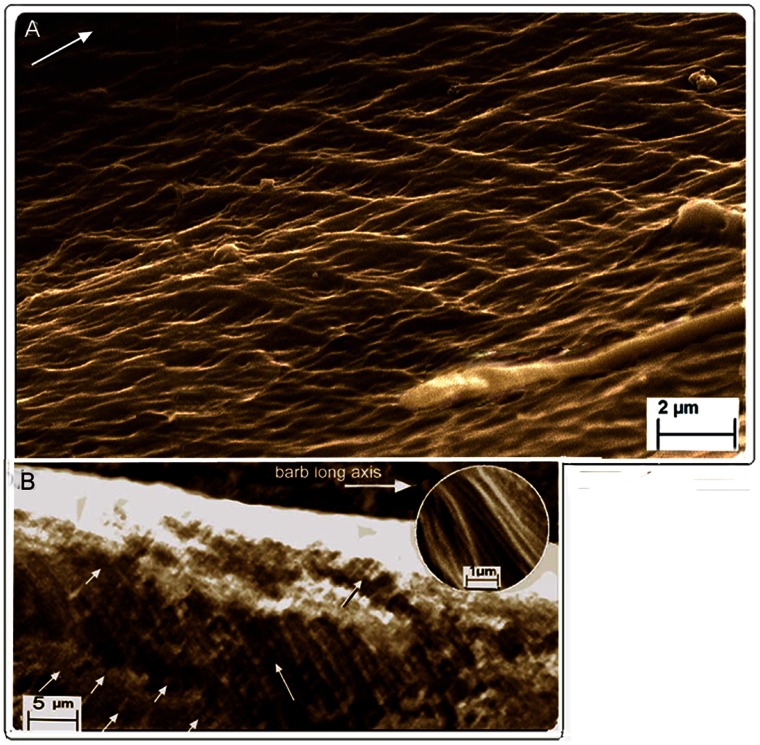
SEM of fungal selective matrix disassembly of barb epicortex in *Gallus gallus*. (A) Alternating cross-fibre structure of epicortex in section just above a barbule and below the cortex. Fungus in bottom right corner shows papulose apical tip of hypha. Arrow = long axis of barb. (B) Longitudinal view of barb from mid-length of rachis and at half-to three-quarters barb length reveals 3-D view of fibril bundles in epicortex *in situ* (∼0.8 µm thick). Fibrils, left and right (latter mostly degraded) oriented at ∼48.24° to the long axis (mean, Table S1). Inset shows detail of fibrils.

Given that fungal selective delineation is a relatively new method of investigation, all findings here, as in the previous study [4], are from both fungal delineations and conventional histological methods (Figs. 1–5 and Figs. S1–S5). Fungi preferentially degraded the amorphous protein matrix of the feathers in a number of samples of both the rachis and barbs (Figs. 1, 2A, 4A, D–E, 5) (Figs. S1A, C–D, S2C–E, S3C), thereby exposing the β-keratin fibres (although out of the scope of the present study a schematic hypothesis of the possible structures biodegraded by the fungi in the amorphous matrix was provided in Figure 5B of a related study [4]. Although all findings here are supported by conventional histological methods on native (non-biodegraded) feathers (Figs. 2B, 3, 4B,C) (Figs. S1B, E, S2A, B, S3A, B, D–G, S4, S5), hindsight wisdom from the fungal degradation experiments was ultimately the driving force for persevering with the conventional methods (requiring perhaps over a hundred histological sections).

### Rachis

A superficial layer of the rachidial cortex is partially exposed by selective fungal degradation. It shows thick fibres in relief, oriented with the rachis long axis and identified as syncitial barbule cells by their characteristic diameter (exposed deep enough to show a diameter of ∼5 µm). The layer is no more than a few cells deep (Fig. 1A and insets) (Fig. S1A) and adds to the layers previously identified [4] i.e. the thickest, which forms about 85% of the depth of the cortex (directly overlying the medulloid pith), comprised of fibres oriented with the rachidial long axis, and above it a layer approximately 15% the depth of the cortex, comprised of fibres oriented at ∼70° to the long axis [4]. All three layers occur in the dorsal and ventral cortex.

An entirely new structural organization of β-keratin fibres is identified by fungal matrix degradation in the lateral walls of the rachis. It is comprised of helices of alternate layers of oppositely oriented fibres approximately 45° to the rachidial long axis (Figs. 1B, C, 2A) (Fig. S2C). While this crossed-helical array of β-keratin fibres dominates the structure of the lateral walls, a few superficial layers of fibres, near the boundary with the cortex, extend parallel to the rachidial long axis and overlie it (Fig. 1C). This new structural architecture of cross fibres occupies the entire area of the lateral walls, from just above and below the barb dorsal and ventral surfaces. A transverse section (Fig. S2B) shows that the cross-fibre structures occupy virtually the entire depth of the lateral walls, except for where the cortex may overlap them near the dorsal and ventral edges. A complete cross-sectional dissection of a native flight feather of *Falco peregrinus* (Fig. 2B) (Figs. S4A, B, S5 (high resolution)) includes the superficial cuticle and shows the entire cross-fibre architecture of approximately 16 two-ply layers. The data from the section are aided by the somewhat “jagged”, sharply oblique cut (cf. smooth right-angled cut in resin-embedded section in Fig. S2B), which helps reveal the alternating crossed-fibre patterns despite the binding matrix. A small section of tissue that had been pulled out slightly in the sectioning process (Fig. 2B, rectangle) (Fig. S4B) shows a two-ply warp and weft of the fibres, which underscores the fabric-like nature of the tissue of the lateral walls. Given the distinctive and novel architecture of the lateral walls of the feather rachis, it is defined here as the epicortex (the prefix epi- means 'near' as e.g. in epicalyx rather than the more frequent meaning of 'above'). The epicortex varies between 15–30 µm thick and in some sections examined can be ±40 µm thick (e.g. Fig. 2B). Fibril angles in most sections are ∼40–50° to the rachis long axis (Table S1). Importantly, one section shows the boundary between the rachidial cortex and epicortex, the latter firmly entrenched under the cortex (Fig. 1C, arrows). An area close to the epicortical surface is identified by traces of the microvillus cuticle (Fig. 2A, right, middle), which is frequently eroded in the more exposed cortex (also see Fig. 2B, top left). Micro-folds in the section (Fig. 2A, bottom) apparently indicate natural surface wear and loss of tension (also indicated by waviness in fibres and low angles) in contrast to straight fibres in a slightly deeper layer (Fig. 2A, inset). As far as we can tell from cross-sections, the epicortex is absent in the dorsal and ventral walls of the rachis.

The plasticity of the rachidial epicortex is demonstrated by a tangential dissection ∼10 µm deep along the rachidial long axis, close (∼2–3 µm) to the medulloid pith layer (Fig. 3A and inset). The section shows the medulloid pith cells from directly below impressed in relief upon the epicortex. The plasticity is clearly facilitated by the cross-fibre architecture of the epicortex (inset), which presumably would be even more striking when the medulloid pith cells are air-filled, as in the native state of feathers, and during flexion of the rachis. Sometimes, it is not the pristine sample but one less than perfect that may allow complex interpretations on morphology and function (e.g. Fig. 2B). Another such example (Fig. 2A) shows the consequences of stress in the epicortex over time (wear), reflected by tensional changes to the cross-fibre architecture and surface micro-folds (foreground).

### Barbs

Several layers of fibres of the rachidial cortex continue on directly to form the dorsal and ventral walls of the barbs (Fig. 4A, B). The fibrous tissue here is 30–40 µm deep (Fig. S3D–F), nearly an order of magnitude smaller than that of the rachis. Significantly, selective fungal disassembly and micro-dissections in *Gallus gallus* and *Falco peregrinus* confirm that the cortex of the barbs and rachis have an identical microstructure i.e., comprised of the characterizing syncitial barbules cells (Fig. 4A, B) (Fig. S1A, inset and S2E, inset). In the barbs, however, they are oriented solely along the long axis and at most comprise 6–7 layers depending on the thickness of the dorsal and ventral cortical layers.

The lateral walls of the barbs are thin, ranging from about 5–10 µm thick (Fig. S3B, D–G). The lateral walls, as in the rachis, are comprised of a 3-dimensional construction of layers of oppositely oriented fibres (approximately 45° to the barb long axis) and are likewise named the epicortex (Figs. 3B, 4C–E, 5) (Fig. S1E, S2D, S3A–G). The epicortex extends the entire area between the dorsal and ventral cortex and includes the area above and below the barbule attachment zones (Fig. S3D, E, F). The entire organization mimics the epicortex of the rachis and clearly appears to be an uninterrupted continuation of the latter. As in the rachidial epicortex, the plasticity of the barb epicortex is demonstrated by impressions upon its surface from the underlying medulloid pith cells (Fig. 4C).

Distally, at approximately three-quarters the barb length, i.e. the slenderest part examined, the fibre helical structure extends over much of the barb height (Fig. 5B), oriented at a mean 48.24° (Table S1) to the barb long axis (traces of oppositely oriented fibrils are also seen in a largely degraded overlying layer. Fibril/fibre bundles of the helical meshwork in both the rachis and barbs range between 0.1–0.8 µm in diameter (Table S2).

Significantly, conventional dissections supplemented most observations from fungal delineations here (Figs. 2B, 3, 4B, C) (Figs. S1B, E, S2A, B, S4A, B, S5) and in the related study [4]. Unquestionably, selective fungal disassembly of the keratin matrix was the most successful of attempts to expose the fibrous texture with respect to revealing both the helical crossed-fibre meshwork and syncitial barbules *in situ* with remarkable 3-dimensional visual clarity. The flight feathers (leading and trailing edges) were tested from the five bird species, domestic chicken, buzzard, falcon, and owls because they occupy different lifestyles (see Material & Methods). We reserve our findings to them but reasonably speculate that the crossed fibre structure of the epicortex and microstructure of the cortex occurs in the flight feathers of birds generally. Contour feathers of *Gallus gallus* also show the same microstructure and one may further speculate that there are no significant differences in feathers generally, perhaps only in differences in relative depth between the two types of microstructures that may be associated with different functions.

We note in parenthesis that having established strong reference bases from conservative methods of feather degradation here and previously [4], future accelerated techniques of fungal degradation [11] as a tool for delineating the microstructure of problematical biological materials are currently being investigated by the first author.

### Summary of Microstructural Findings

(1) The rachidial cortex is comprised of syncitial barbule cells, the majority, which occupies ∼85 percent of the cortical depth, are oriented with the rachidial long axis and located directly above the medulloid pith [4]. Above this is a layer of syncitial barbule cells, comprising ∼15percent of the cortical depth, oriented uni-directionally at ∼70° to the rachidial long axis [4]. Here, a new superficial layer of syncitial barbule cells a few layers thick are aligned with the rachidial long axis.

(2) The lateral walls of the rachis (the epicortex), are considerably thinner than the cortical walls and are characterized by a novel microstructural organization of multiple layers of oppositely oriented fibres (the cortex may overlap the epicortex slightly at the dorsal and ventral edges).

(3) The barb possesses a cortical layer dorsally and ventrally and, as in the rachis, is characterized by syncitial barbule cells, which, however, are oriented solely along the barb long axis.

(4) The lateral walls of the barbs (the epicortex) are much thinner compared with the cortex and have a cross-fibre architecture identical with that of the rachidial epicortex.

## Discussion

For about 150 years the syncitial barbule cells were known solely as free barbules and in neonatal feathers until it was shown that they form the highest structural organization of fibres of the feather rachidial cortex [4]. Here, again contrary to histodifferentiation studies [2], [3], syncitial barbule cells are identified in the barb cortex, perhaps from an anatomical perspective somewhat surprisingly given the relative thinness of the cortex compared to that of the rachis. Functional implications of syncitial barbule cells in the barb cortex may be considered essentially similar to that previously proposed for the rachis [4].

Given that previous studies treated the lateral walls of the barbs and rachis as simply thinner versions of the dorsal and ventral walls, we consider a novel architecture of inextensible crossed-fibres in the lateral walls (epicortex) functionally highly significant. The proven mechanical importance of this design architecture in other animals (see below) is considered critical in understanding its mechanical significance in the feather rachis and barbs because it involves a surface area that is approximately equal to that of the cortex (perhaps greater in the barbs; see e.g. Fig. S3D, E, F) i.e. the cross-fibre architecture occupies at least half the surface area of the feather.

The cross-fibre microstructure described needs some clarification. It is important not to confuse it with findings by X-ray diffraction analyses that suggest an anisotropic fibre structure of the feather rachis [12]. Based on their x-ray diffraction study, Earland et al. (12) proposed that the feather calamus was “polyphase in structure, consisting of an interior layer with molecular orientation along the axis, and an exterior layer with orientation at right angles to the axis” i.e. that the “calamus is composite.” This of course does not imply a meshwork but it is part of a mechanical design strategy to reinforce predominantly longitudinally oriented fibres that are prone to splitting along the long axis i.e. failure by the Cook–Gordon mechanism–by incorporating a thin outer layer of tangentially oriented fibres [13]. This cortical description in the rachis is modified here to include at the surface one or two layers of syncitial barbule cells aligned with the long axis of the rachis, possibly for better aerodynamics (similarly in the barbs). Two things are, however, comparable in Earland et al.s’ [12] and Lingham-Soliar et al.s’ [4] findings, the depth of the outer layer of approximataly 15% the total cortical depth and the inclination of the fibres at approximately 70° to the rachidial long axis. Interestingly, in the context of selective biodegradation here, Earland et al. [12] intuitively raised doubts based on their X-ray diffraction patterns of chemical fractions as to whether or not native feather keratin is in an exclusively β-configuration and suggested the possibility that it contains some α-protein [6], [7].

We note in parenthesis that x-ray diffraction studies on the feather have concentrated to date on the rachis (see e.g. references in [4]), in particular the dorsal and ventral walls (cortex), which is understandable for two reasons, first, it is “uncluttered” by the hundreds of barbs found in the epicortex and second, there was no reason to think, until now, that the lateral walls were any different from the dorsal and ventral, other than in thickness. Consequently, there are no x-ray data to our knowledge on the cross-fibre architecture of the epicortex.

Bodde et al. (14) correlated the fibro-microstructure of the feather [4] with biomechanical investigations, “The longitudinally oriented fibres revealed by Lingham-Soliar et al. (2009 [online]) to be syncitial barbules cleave in a brittle fashion, while the more superficial, tangentially oriented fibres at the cuticle seem to fail by ductile tearing.” Although they tested the rachidial cortex their ductile tearing tests may have involved the boundary with the epicortex (Fig. 1C). With respect to our findings on the epicortex, we believe a design in which barbs and barbules are anchored to the rachis and barbs respectively by a meshwork of cross-fibres seems not just reasonable but, with respect to mechanical principles, predictable if dangerous ductile tearing [14] is to be minimized during bird flight. Indeed, a similar meshwork of fibres has been shown in other animals where control surfaces such as fins are anchored to the body to withstand extreme stresses during locomotion [15], [16], [17].

**Figure 6:**
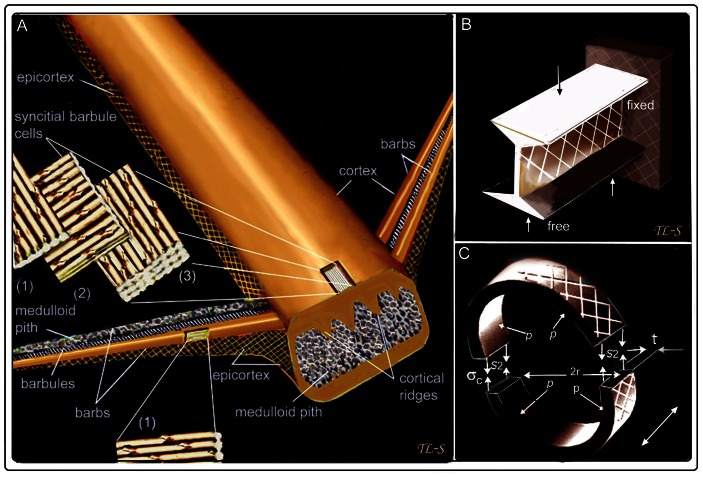
A new microstructural fibre model of feather rachis and barbs and classic engineering analogues. (A) An exploded view of three fibre divisions of the rachidial cortex and one of the barb cortex (both in dorsal and ventral walls). The cortex is identified by the thick syncitial barbules cells (∼6–8 µm in diameter [4]). The lateral walls of the rachis and barbs, the epicortex, are characterized by a crossed-fibre structure and absence of syncitial barbules cells. One barb shows cortex removed to expose the medullary pith cells. (B) Diagrammatic view of rachis and barb as an I-beam (here, a cantilever) in which most material is concentrated in the upper (tension) and lower (compression) surfaces to resist maximum stresses –with the “web” in the middle to resist shearing forces at ±45°. (C) Diagrammatic view of barb as a thin-walled pressure cylinder. Slice in latter shows circumferential stress is twice the longitudinal stress i.e. *S_2_ = rp/t*. *S* = stress, *t* = thickness, *p* = pressure, *r* = radius. Double-headed arrow = long axis of cylinder (modified after Lingham-Soliar [17]).

We believe a new microstructural fibre model for flight feathers is warranted (Figure 6).

### A New Fibre-Microstructural Model for Flight Feathers

Lingham-Soliar et al. [4] revealed a novel architecture in the feather rachis of β-keratin syncitial barbule cells (6–8 µm thick) “glued” together to form the rachis. Binding of fibres into bundles by a glue or matrix compares with an engineering principle developed by materials researchers to minimize lateral slippage and increase toughness 18. The syncitial nodes are thought to improve cutting energies, increase resistance to fracture e.g. propagation of a crack and prevent fibre “pull-out” [4]. New findings here allow a more complete picture of the fibre architecture of the feather. The barb cortex is structurally similar to that of the rachis, comprised of syncitial barbule cells. The main microstructural findings here, however, involve the epicortex of the rachis and barbs. The epicortex is comprised of a fibre structural system previously unknown in keratin–a helical crossed fibre architecture, which is known to have profound mechanical implications in nature and engineering [19], [20] (below). We consider it appropriate therefore to briefly discuss the functional implications of these novel microstructural characteristics of the feather barb and rachis.

### Biomechanical Implications of the Crossed-Fibre-Microstructure of the Feather

Unfortunately, there are at present few studies on the mechanics of feather barbs. Butler and Johnson [21] performed breaking stress experiments to determine whether melanised feather barbs are stronger than un-melanized, the only mechanical experiments to our knowledge on individual feather barbs to date, and Ennos et al. [22] performed mechanical tests on the feather vane. Our new model of the cross-fibre microstructural design in the feather we believe may lead to engineering explanations as e.g. did seminal findings on the cross-fibre microstructure of shark skin fibres [23], [24]. At the heart of our functional interpretations is the hypothesis of a geodesic crossed fibre system of the integument, which was first presented by Clark and Cowey [25] in an elegant anatomical and mathematical study to explain changes in extensibility in nemerteam and turbellarian worms. Since their classic study the model of a thin-walled, fibre reinforced cylinder (Fig. S3I (iii)) was applied to a number of biological systems, initially mostly in invertebrates [26]–[29] but later in vertebrates, principally marine forms [15], [16], [17], [23], [24], [30], [31] and more recently in fossils animals [32]–[34]. The crossed fibre architecture of the integument of animals is now firmly entrenched as a design principle of biomechanics [35](see [36] for an excellent review).

In engineering terms, the rachis and barbs of the feather may be compared with the I-beam i.e., a structure with thickened upper and lower walls to resist longitudinal tension and compression stresses and an intervening cross-fibre microstructure at ±45° to the long axis to resist vertical or shearing stresses (Fig. 6B). A second engineering model based on the thin-walled cylinder [19], [20] is perhaps an even closer analogue (Fig. 6C). We will concentrate on the barb in which some relevant calculations have been made [21] although most conditions we suggest would apply equally to the rachis.

Calculations on the ratio of wall thickness to mean radius in the barbs of flight feathers vary because of an elliptical cross-section (as in some worms [25]) but reasonably satisfy the criteria of being thin walled (radius *r* is larger than 5 times its wall thickness) [21] and of possessing a deep hollow centre (or equivalent). The barb possesses two additional structural features. First, the thin external epicortex is reinforced by layers of oppositely oriented crossed-fibres. In natural and engineering systems failure in a thin-walled, hollow construction usually occurs by splitting at 45° to the long axis because the circumferential stress is twice the longitudinal stress [19], [20] (Fig. 6C), stresses that may be a consequence of increased internal pressure or strong bending or twisting movements [22], [24]. Second, the barb, rather than possessing a hollow center, has a foam core (medulloid pith, Fig. S3B, D, E, F), a feature that was found to optimize or improve the load ratio and moment ratio over an equivalent hollow shell [37].

The thicker dorso-ventral walls of the barbs are considered to increase flexural stiffness during flight by allowing for twisting when loaded with dangerously high forces, by avoiding failure by bending and, complete failure by buckling rather than rupturing (21). Explanations, such as allowing twisting when loaded with dangerously high forces, may be helped by the microstructural data of the feather rachis and barbs as presented here. Whereas predominant axial orientation of the fibres maximizes flexural rigidity while minimizing wing inertia and drag [38], the cross-fibre microstructure of the epicortex (comprising about half the surface area of the feather) is consistent with biomechanical proposals that anisotropy of fibre orientation is an adaptation to allow torsion of the asymmetric feather vane [22]. During passive twisting or flexion of the barb, large lateral contractions of the fibres are incurred because of tension on the convex barb surface (Fig. S3I (ii)). We consider the cross-fibre system a key mechanism for preventing damage to the barb by increasing the amount of flexural strain it can withstand. Consequently, a system that enables the barb to twist smoothly without kinking or splitting and to resist torsional deformation should help prevent or delay the onset of buckling during flight. Thus, we believe that functionally the advantage of lower transverse tensile strength that allows the rachis and barbs to bend smoothly greatly outweighs the disadvantage of lower flexural rigidity (longitudinal strength) [13].

The cross-fibre microstructure of the epicortex we believe would also enable it to behave comparable to a thin-walled pressure cylinder in the control of hoop and longitudinal stresses. However, perhaps the final word with respect to the cross-fibre microstructure must be the crucial role it plays in anchoring the barbs and barbules to the rachis and barbs respectively and in minimizing ductile tearing as a consequence of the significant stresses involved during bird flight.

An understanding of how “springs” incorporated in the integument of animals influence the mechanical behavior of locomotory structures is demonstrated at present in only a few studies particularly with respect to their integration in biomechanics, kinematics, hydro- and aerodynamics and metabolism [17], [39]. A number of conditions described above suggest that the feather may function as a spring. For example under compressive loading, transference of tensile stresses from the cortex and epicortex to the medulloid pith (Figs. 3A, 4C, 6A) (Figs. S1E, S3B, D, E, F and S4C) and the latter's ability to absorb high amounts of energy [38], [40], [41] may be important in e.g. restoring barbs and rachises to their normal position following torsion or flexion, and at the gross level, in decelerating the wings at the end of the downstroke [42].

### Evolutionary Comments

As stated in the related study [4], the evolution of the feather is a contentious subject with two highly polarized hypotheses,”the classical model is that feathers evolved from reptilian scales (Maderson 1972)–that a basic rachis would have formed first… then barbs and finally barbules. An alternative hypothesis is that barbs form first [in the context of barbs and rachis] during development, and the rachis, a specialized form of fused barbs, appeared later as an evolutionary novelty (Prum 1999; Yu et al. 2002).” The independent findings here may be significant from an evolutionary perspective because they present morphological evidence that the syncitial barbule cells, largely indistinguishable from their free form, are found as a major structural component of, in addition to the rachis [4], the feather barbs. This new finding further opens up the question, did syncitial barbule cells appear fully formed in the ancestral rachis as part of a functional structure, subsequently contributing to barbs and barbules–or, did they evolve gradually from relatively simple undifferentiated barbule-like structures to specialized free barbules involved in thermo-regulation, later coopted to maintaining feather vane integrity [43] and finally, to becoming involved as internal structural units of the cortex of the barbs and eventually that of the rachis, with the original characters adapted to new mechanical roles [4]?

### Future Directions

Fungal selective matrix degradation in the feather barbs of birds examined here has proved important in resolving vital new microstructural characteristics of the feather, as in a previous investigation of the rachidial cortex [4], albeit this time with respect to resolving a much finer fibril microstructure, in some cases by a factor of 100 (Fig. 4, Table S2). The method has the advantage of being *in situ* and of creating minimal distortion as e.g. when slicing through fine fibres and destroying the 3-dimensional microstructure and, quite significantly, in providing the most visually distinctive images.

From a functional perspective feather microstructure has been understudied and consequently underestimated. It is hardly surprising therefore that one of the most remarkable structures in nature has contributed so little to the field of biomimetics [44]. Dispite the highly conserved molecular structure of feather β-keratin, its inherent capacity for intracellular, hierarchical self-assembly [4], [45] appears quite remarkable. The present study, as in the previous [4], demonstrates this capacity–this time in the classic crossed fibre architecture, previously unknown in keratin, which we consider crucial to feather biomechanics. As well as adding to our understanding of questions related to bird flight, our increasing knowledge of self-assembling β-keratin fibres may provide inspiration for new generations of nanofibres in the field of biomimetics [44]. We hope our use of microbes as an investigative tool may also provide inspiration for analysing the microstructure of other keratins and perhaps other problematical natural materials.

## Materials and Methods

### Ethics Statement

“N/A.” No permits were obtained because no birds were harmed in the course of this study. “All feathers were freshly moulted and provided by Umgeni Bird Park, Durban and Rainbow Chickens, Pietermaritzburg”.

Rachises and barbs of flight feathers of the birds involved in the study came from captive jackal buzzard, *Buteo rufofuscus*, white-faced owl, *Otus leucotis*, spotted eagle owl, *Bubo africanus*, peregrine falcon, *Falco peregrinus*, (Umgeni Bird Park, Durban) and the domestic chicken, *Gallus gallus* (Rainbow Chickens, Pietermaritzburg). The domestic chicken is more or less a standard, the falcon and buzzard are swift diurnal predatory birds and the owls are nocturnal predators with a “softer” flight plumage.

### Experimental Selective Degradation

Both fresh feathers and fungal treated feathers of *Gallus gallus*, *Falco peregrinus*, *Otus leucotis* and *Bubo africanus* were tested. Detailed methods of our fungal degradation of feathers may be found in the related previous study (Material and Methods including Supplementary Information [4]). To promote fungal degradation in the present study, feathers of *G. gallus*, *F. peregrinus*, *O. leucotis* and *B. africanus* were placed in a plastic tray containing a layer of feathers of *G. gallus* with known high fungal infestation [4] and covered by another layer of fungal infested feathers (predominantly *Altenaria* sp.; see culturing and identification by rRNA analysis [4]) and incubated for 9 months. The temperature of incubation was maintained at 22°C and humidity at 50% [4]. Consistent with the previous emphasis on natural biodegradation techniques [4], no artificial means (e.g. increased moisture, temperature or catalysts) were used to accelerate the fungal degradation process, which we consider important given the finer levels of fibre structural delineation in the present study compared to the previous [4].

### Standard Histological Preparations

Besides fungal delineations of feathers, the rachis and barbs were dissected or peeled (under a binocular microscope) in attempts to reveal the fibre microstructure by more conventional means. Tangential sectioning (parallel to the surface) for the helical cross-fibre structure was difficult for reasons explained previously [4] but even further exacerbated here with respect to the much smaller dimensions of the barbs compared to the rachis. Tangential sections were obtained, which critically required more than one fibre layer (involving barb dimensions of microns and nanometers) so as to establish cross-fibre layers. Cross-sections of entire barbs were made near their attachments with the rachis, with and without resin embedding and etching (see [4]).

### Microscopy

Sections were examined predominantly by scanning electron microscopy although a few were examined by transmission electron microscopy, which proved generally ambiguous for micro- and nanometre levels of structural organization (as opposed to molecular). Furthermore, they could usually only be interpreted with hindsight knowledge from the SEMs [4]. Examinations were of representative areas of the barbs, selected along the length of the rachis and along different points of the individual barb (from approximately three-quarters length from base and tip of rachis and barb respectively).

## Supporting Information

Figure S1 **SEM of rachis cortex and epicortex microstructure.** (A, C, D) shows fungal surface degradation of *Gallus gallus* cortex and (B) and (E) dissections of native (non-biodegraded) feathers. (A) Syncitial barbules in relief at the rachidial surface (identified by diameter). Rectangle shows a syncitial barbule removed from the barb cortex by fungi. Circular inset of fibres of syncitial barbule delineated by fungi. (B) Rachidial epicortex of *Gallus gallus*. Cross-fibres just above the line of the barbs. (C, D) Surface of rachidial cortex. (E) Barb of *Bubo africanus*. Tangential section. Fibres oriented about 45 degrees to the rachis long axis (arrow) close to the medulloid pith (in relief). Fibres are closely packed but impressions of underlying fibres can be detected in places. Raised oval area is from pressure from medullary pith cell (Arrow = long axis of section).
(TIF)

Figure S2 **SEM of rachis and barb microstructure.** (A) Dissection of native (non-biodegraded) feathers, resin embedded and etched. *Buteo rufofuscus*. Tangential/longitudial section of rachis epicortex at the rachis-barb interface (leading-edge). The top layer of fibres has been largely sheared off. Top right shows thicker fibres bundles. Inset, fibril angles approximating those in the section; arrow indicates longitudinal axis of rachis. (B) Native (non-biodegraded) *Gallus gallus*. Transverse section (cut at right angles to the rachis long axis) of epicortex of rachis, adjacent to barb. About 15 fibril layers are exposed (r = lateral or angled view of fibres and tr = full transverse view). Arrows show some radial fibres. Fungal selectively disassembled matrix (C–E). (C) *Gallus gallus* rachidial epicortex. (D) *Bubo africanus*, barb epicortex close to ventral surface, showing oppositely oriented fibres. (E) *Falco peregrinus* barb. Far left, two degraded syncitial barbules lifted of the barb cortex by fungal activity. Barb cortex surface (demarked by curved line) shows ridges indicating syncitial barbules (double-headed arrows). Cortical fibres oriented along barb long axis (fungi, top left and centre. Far right, epicortex. Inset, degraded barb of *Bubo africanus* showing syncitial barbules. Arrow = barb long axis.
(TIF)

Figure S3 **Barb anatomy, microstructure and dynamics.** (A) Dissection (tangential) of native (non-biodegraded) barb epicortex of *Falco peregrinus* showing two layers of oppositely oriented fibres. (B) *Otus leucotis*. Dissection (transverse) of native (non-biodegraded) barb adjacent to rachis showing layers of oppositely oriented fibres. (C) Distal part of a barb, lateral view of epicortex (top, wide view, bottom close-up), fungal degraded. Fungi have created windows to an inner layer of cross-fibres. Black arrows indicate superficial thicker fibres oriented with the barb long axis. (D–F) Native (non-biodegraded) cross-sections of barb cut close to rachis of *Gallus gallus*, *Falco peregrinus* and *Bubo africanus* respectively, resin embedded and etched. (G) Native (non-biodegraded) barb of *Gallus* gallus cut longitudinally to show the epicortex and medulloid pith. (H), TEM of 3 layers of fibrils in rachis epicortex in radial (R), and transverse (T) orientations (radial view, fibrils thicker and sausage-shaped). (I) Diagramatic representation of helical cross-fibre dynamics. (i) Fibres at rest (large and small arrows–fibres along bias and weft resp.). (ii) bending and extension of the helical fibril structures of the rachis and barb. Lateral contraction causes the fibres on the convex side to become stretched (high Poisson’s ratio) because of an increase in the fibre angle and tension. (iii) Clark and Cowey’s [25] model was based on the idea of a fibre reinforced cylinder. The relationship between volume and fibre angle can be shown in the included equation. The curve in the graph represents the theoretical relationship between the volume contained by the fibre system and the inclination of the fibre to the longitudinal axis. With extension (as in a worm), the section diameter and fibre angle both decrease and conversely, with segment shortening the fibre angle and segment diameter increase. The horizontal line represents the constant volume of *Amphiporus lactifloreus* (worm). It intersects the curve at F and G, which are the limiting positions of elongation and contraction, respectively, for the species. Formula: D = the length of a single turn of the geodesic fibre bounding the section, *θ* = the angle between the fibre and longitudinal axis.
(TIF)

Figure S4 **Rachis anatomy and microstructure. Native (non-biodegraded).** (A) Detail of epicortex in text figure 2B. (B) Detail of epicortex section from text figure 2B showing 2-ply of fibre warp and weft.(C, D) *Gallus gallus*. Cross-sections of rachis (mid-length). (D) Diagrammatic representation of transversely dissected rachis. Scale bar: (A) = 2 µm; (C) = 100 µm; (D) = 50 µm.
(TIF)

Figure S5 **Rachis epicortical microstructure of native (non-biodegraded) feather of** ***Falco peregrinus*** **.** For full legend see text-figure 2B.
(TIF)

Table S1 **Fibre angles (degrees) in feather epicortex in four bird species.**
(DOCX)

Table S2 **Fibre diameter (nm) in epicortex (crossed fibres) of rachis and barbs.**
(DOCX)
