# Supplementary figures and images for: A New Helical Crossed-Fibre Structure of β-Keratin in Flight Feathers and Its Biomechanical Implications
Source: PLoS One. 2013 Jun 10;8(6):e65849. doi: 10.1371/journal.pone.0065849 (PMC3677936; doi:10.1371/journal.pone.0065849)

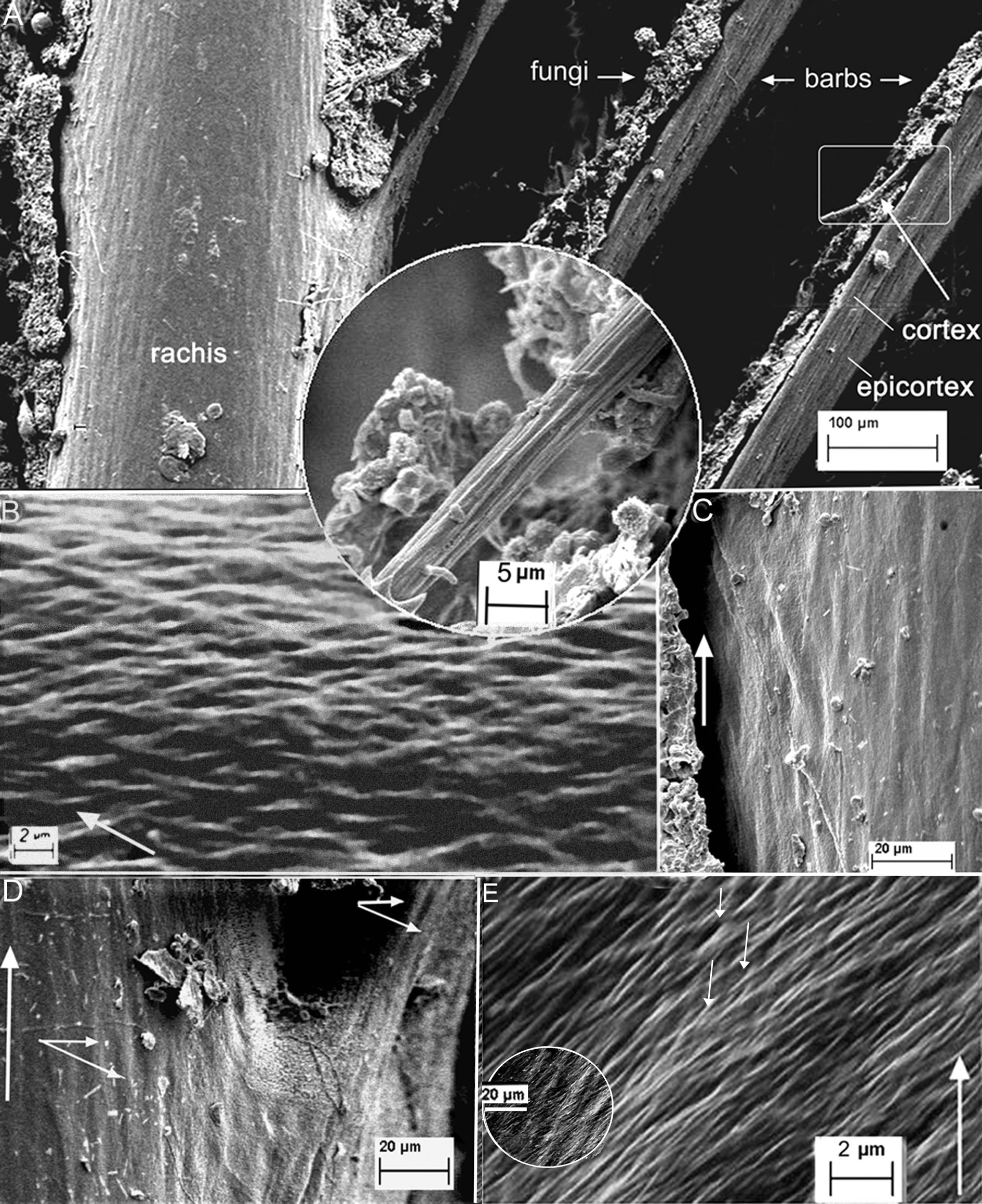

Supplement: Figure S1 — SEM of rachis cortex and epicortex microstructure. (A, C, D) shows fungal surface degradation of Gallus gallus cortex and (B) and (E) dissections of native (non-biodegraded) feathers. (A) Syncitial barbules in relief at the rachidial surface (identified by diameter). Rectangle shows a syncitial barbule removed from the barb cortex by fungi. Circular inset of fibres of syncitial barbule delineated by fungi. (B) Rachidial epicortex of Gallus gallus. Cross-fibres just above the line of the barbs. (C, D) Surface of rachidial cortex. (E) Barb of Bubo africanus. Tangential section. Fibres oriented about 45 degrees to the rachis long axis (arrow) close to the medulloid pith (in relief). Fibres are closely packed but impressions of underlying fibres can be detected in places. Raised oval area is from pressure from medullary pith cell (Arrow = long axis of section). (TIF) [file pone.0065849.s001.tif]

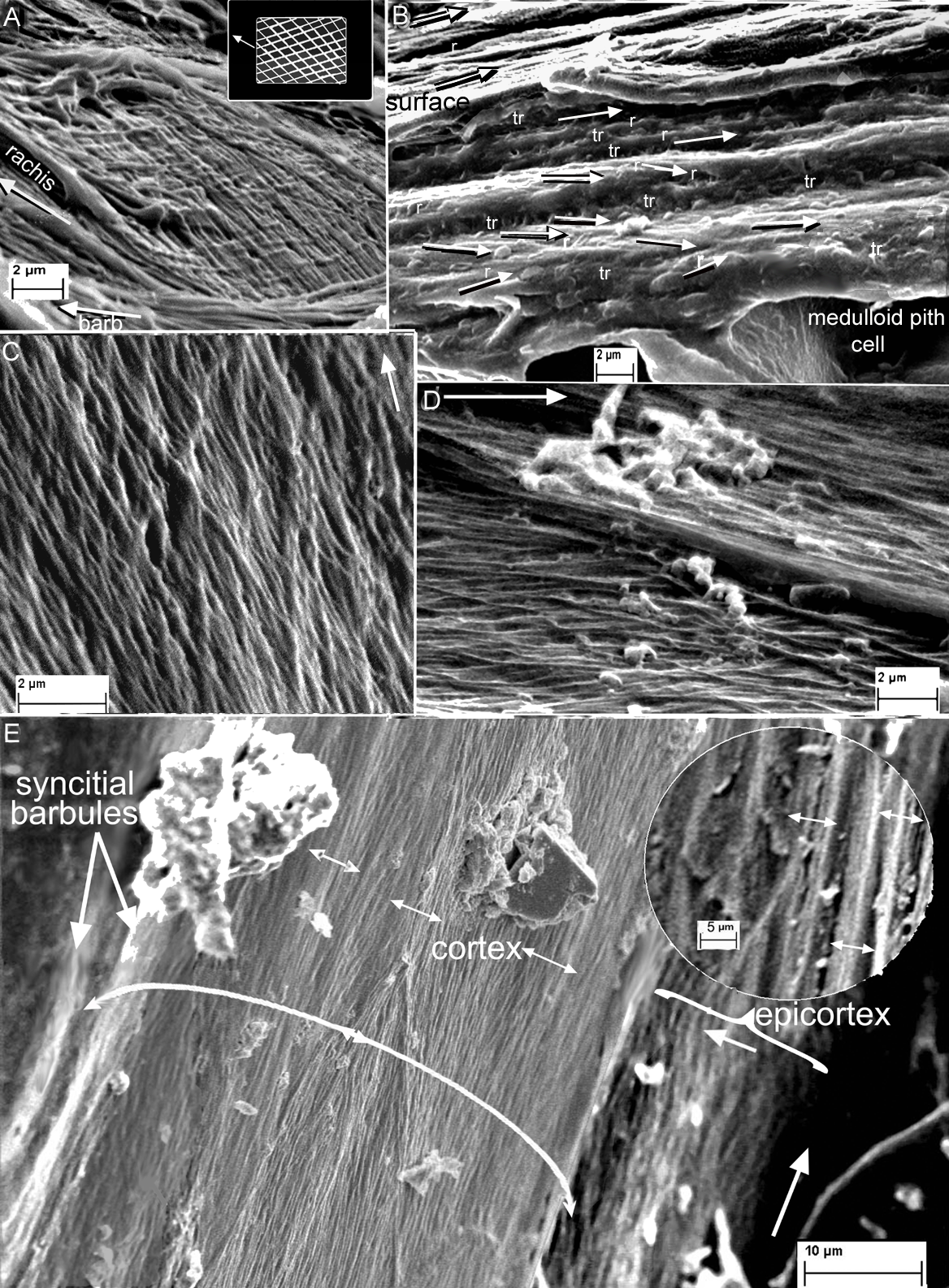

Supplement: Figure S2 — SEM of rachis and barb microstructure. (A) Dissection of native (non-biodegraded) feathers, resin embedded and etched. Buteo rufofuscus. Tangential/longitudial section of rachis epicortex at the rachis-barb interface (leading-edge). The top layer of fibres has been largely sheared off. Top right shows thicker fibres bundles. Inset, fibril angles approximating those in the section; arrow indicates longitudinal axis of rachis. (B) Native (non-biodegraded) Gallus gallus. Transverse section (cut at right angles to the rachis long axis) of epicortex of rachis, adjacent to barb. About 15 fibril layers are exposed (r = lateral or angled view of fibres and tr = full transverse view). Arrows show some radial fibres. Fungal selectively disassembled matrix (C–E). (C) Gallus gallus rachidial epicortex. (D) Bubo africanus, barb epicortex close to ventral surface, showing oppositely oriented fibres. (E) Falco peregrinus barb. Far left, two degraded syncitial barbules lifted of the barb cortex by fungal activity. Barb cortex surface (demarked by curved line) shows ridges indicating syncitial barbules (double-headed arrows). Cortical fibres oriented along barb long axis (fungi, top left and centre. Far right, epicortex. Inset, degraded barb of Bubo africanus showing syncitial barbules. Arrow = barb long axis. (TIF) [file pone.0065849.s002.tif]

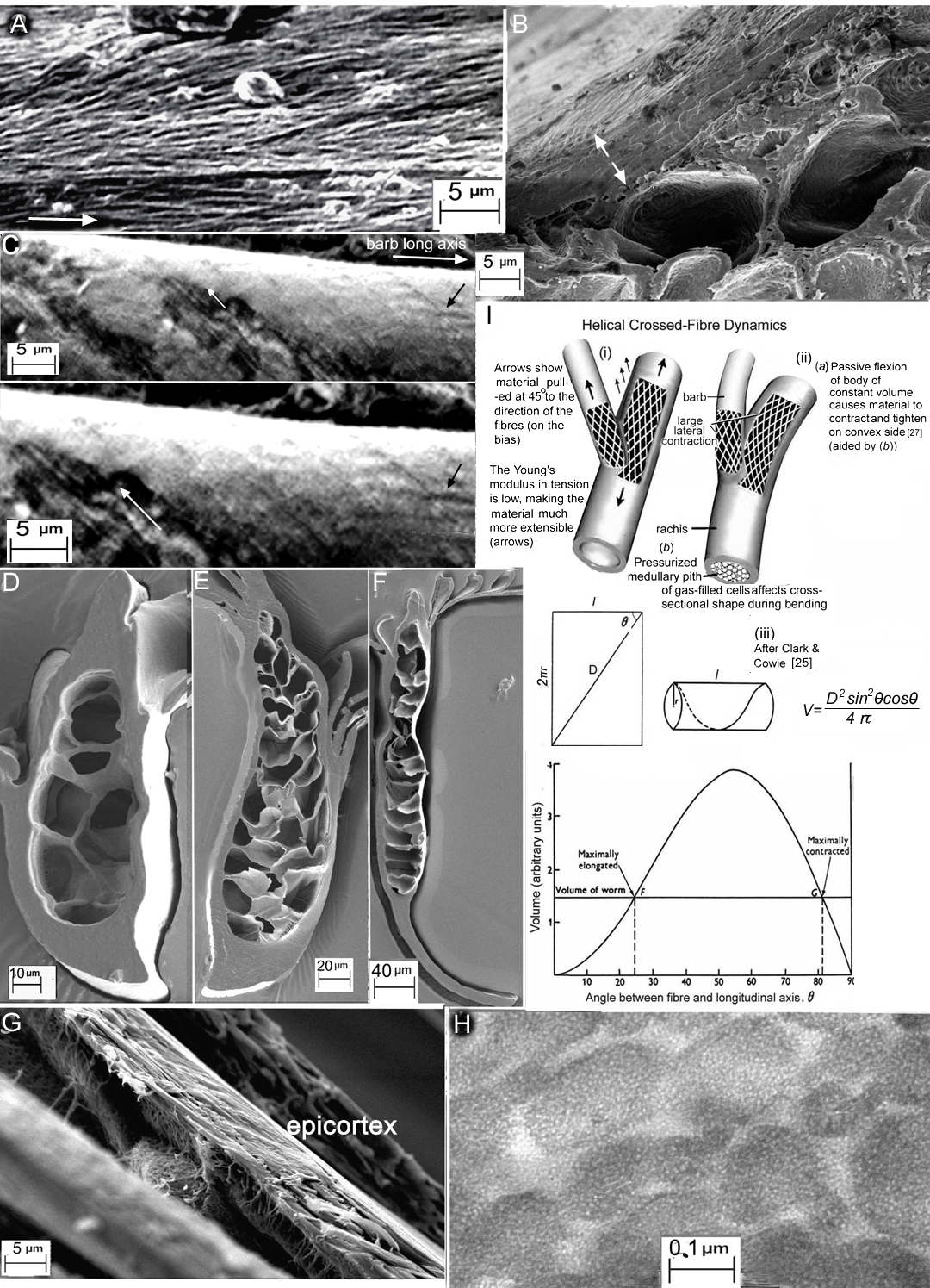

Supplement: Figure S3 — Barb anatomy, microstructure and dynamics. (A) Dissection (tangential) of native (non-biodegraded) barb epicortex of Falco peregrinus showing two layers of oppositely oriented fibres. (B) Otus leucotis. Dissection (transverse) of native (non-biodegraded) barb adjacent to rachis showing layers of oppositely oriented fibres. (C) Distal part of a barb, lateral view of epicortex (top, wide view, bottom close-up), fungal degraded. Fungi have created windows to an inner layer of cross-fibres. Black arrows indicate superficial thicker fibres oriented with the barb long axis. (D–F) Native (non-biodegraded) cross-sections of barb cut close to rachis of Gallus gallus, Falco peregrinus and Bubo africanus respectively, resin embedded and etched. (G) Native (non-biodegraded) barb of Gallus gallus cut longitudinally to show the epicortex and medulloid pith. (H), TEM of 3 layers of fibrils in rachis epicortex in radial (R), and transverse (T) orientations (radial view, fibrils thicker and sausage-shaped). (I) Diagramatic representation of helical cross-fibre dynamics. (i) Fibres at rest (large and small arrows–fibres along bias and weft resp.). (ii) bending and extension of the helical fibril structures of the rachis and barb. Lateral contraction causes the fibres on the convex side to become stretched (high Poisson’s ratio) because of an increase in the fibre angle and tension. (iii) Clark and Cowey’s [25] model was based on the idea of a fibre reinforced cylinder. The relationship between volume and fibre angle can be shown in the included equation. The curve in the graph represents the theoretical relationship between the volume contained by the fibre system and the inclination of the fibre to the longitudinal axis. With extension (as in a worm), the section diameter and fibre angle both decrease and conversely, with segment shortening the fibre angle and segment diameter increase. The horizontal line represents the constant volume of Amphiporus lactifloreus (worm). It intersec [file pone.0065849.s003.tif]

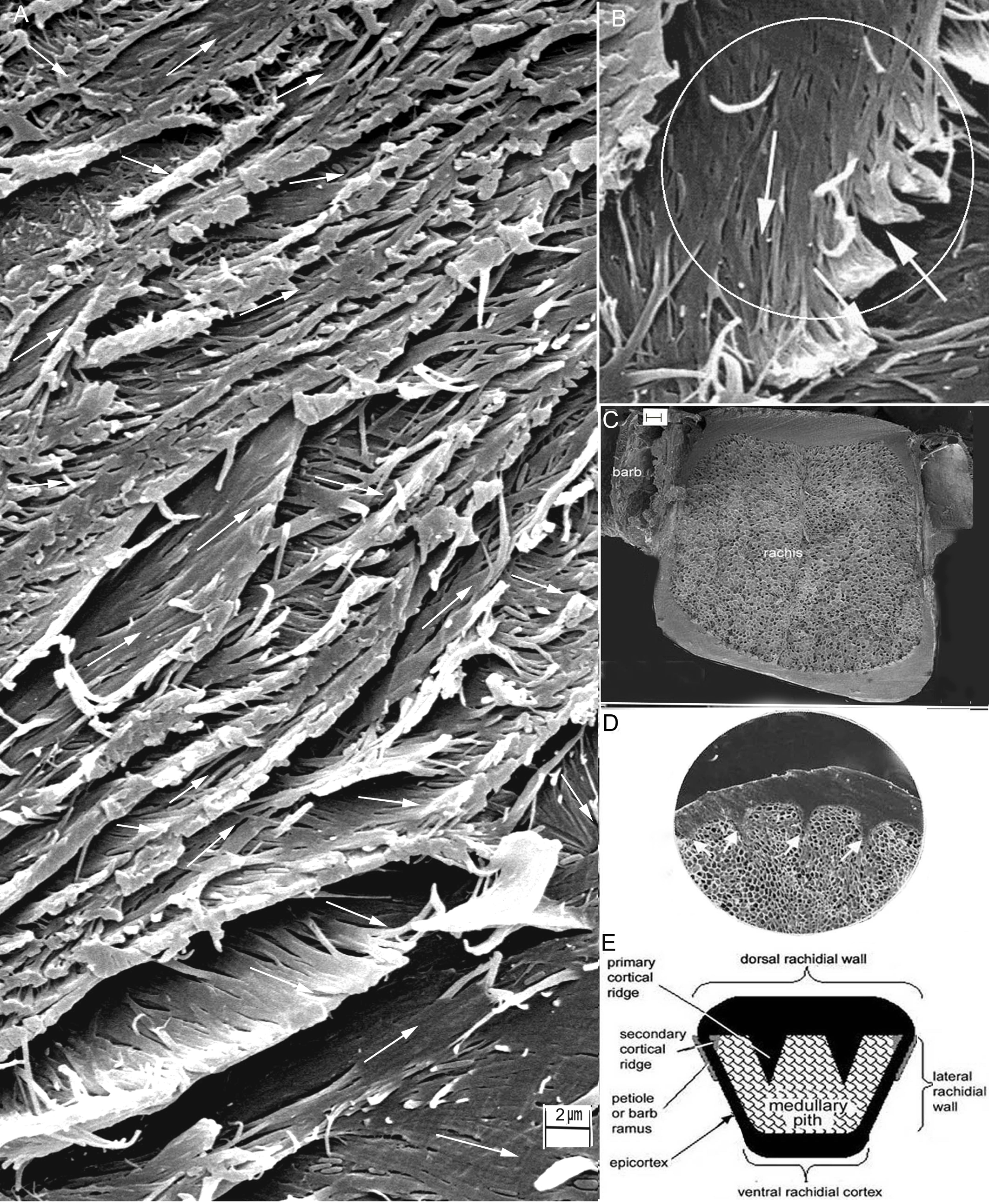

Supplement: Figure S4 — Rachis anatomy and microstructure. Native (non-biodegraded). (A) Detail of epicortex in text figure 2B. (B) Detail of epicortex section from text figure 2B showing 2-ply of fibre warp and weft.(C, D) Gallus gallus. Cross-sections of rachis (mid-length). (D) Diagrammatic representation of transversely dissected rachis. Scale bar: (A) = 2 µm; (C) = 100 µm; (D) = 50 µm. (TIF) [file pone.0065849.s004.tif]

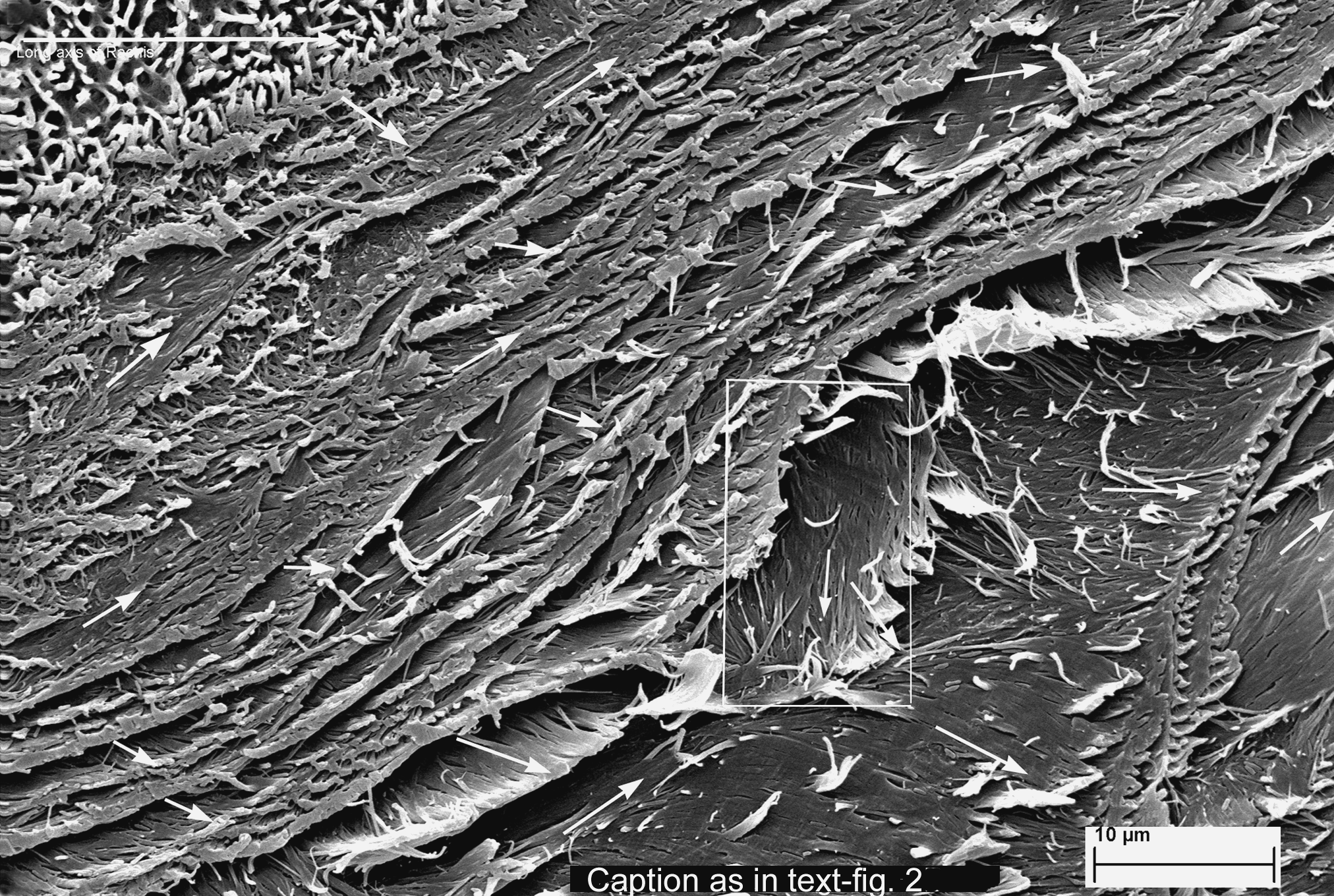

Supplement: Figure S5 — Rachis epicortical microstructure of native (non-biodegraded) feather of Falco peregrinus . For full legend see text-figure 2B. (TIF) [file pone.0065849.s005.tif]
