# Supplementary material for: A New Helical Crossed-Fibre Structure of β-Keratin in Flight Feathers and Its Biomechanical Implications
Source: PLoS One. 2013 Jun 10;8(6):e65849. doi: 10.1371/journal.pone.0065849 (PMC3677936; doi:10.1371/journal.pone.0065849)
Supplement: Table S1 — Fibre angles (degrees) in feather epicortex in four bird species. (DOCX) [file pone.0065849.s006.docx]

Table S1. Fibre angles in degrees in feather epicortex in four bird species.

|  | Barb | Barb | Rachis | Barb | Rachis |
| --- | --- | --- | --- | --- | --- |
|  | *Gallus gallus* F4 | *Bubo africanus* F3 | *Otus leucotis* SI F1 | *G. gallus* ½-¾ barb length | *B. rufofuscus* |
| Mean | 38.37 | 42.25 | 44.83 | 48.24 | 51.53 |
| Std. Dev. | 2.18 | 5.18 | 7.58 | 7.17 | 5.6 |
|  | 40.33 | 41.27 | 50.17 | 41.32 | 45.43 |
|  | 42.44 | 41.7 | 52.25 | 38.84 | 41.58 |
|  | 37.8 | 43.08 | 48.55 | 48.3 | 48.02 |
|  | 36.09 | 48.49 | 44.33 | 47.55 | 51.53 |
|  | 38.48 | 42.08 | 33.7 | 49.42 | 57.02 |
|  | 36.48 | 54.16 | 55.31 | 47.2 | 57.39 |
|  | 36.5 | 43.25 | 48.19 | 53.61 | 55.47 |
|  | 38.8 | 36.61 | 39.89 | 55.34 | 49.95 |
|  |  | 43.83 | 32.67 | 51.82 | 50.34 |
|  |  | 41.35 | 43.24 | 56.77 | 58.56 |
|  |  | 34.61 |  | 40.72 |  |
|  |  | 37.29 |  | 49.75 |  |
|  |  | 38.32 |  | 48.67 |  |
|  |  | 42.45 |  | 42.15 |  |
|  |  | 42.23 |  | 45 |  |
|  |  | 44.94 |  | 44.58 |  |
|  |  | 45.2 |  | 51.33 |  |
|  |  | 37.18 |  | 49.24 |  |
|  |  | 41.68 |  | 52.43 |  |
|  |  | 45.47 |  |  |  |
