# Supplementary material for: A New Helical Crossed-Fibre Structure of β-Keratin in Flight Feathers and Its Biomechanical Implications
Source: PLoS One. 2013 Jun 10;8(6):e65849. doi: 10.1371/journal.pone.0065849 (PMC3677936; doi:10.1371/journal.pone.0065849)
Supplement: Table S2 — Fibre diameter (nm) in epicortex (crossed fibres) of rachis and barbs. (DOCX) [file pone.0065849.s007.docx]

Table S2. Fibre diameter (nm) in epicortex (crossed fibres) of rachis and barbs.

| *Gallus gallus* | **n** | **mean** | **SD** |
| --- | --- | --- | --- |
| Thick fibres (barb) | 53 | 797.50 | 105.6 |
| Medium fibres (rachis) | 50 | 251.98 | 23.57 |
| Thin fibres (barb) | 50 | 101.97 | 24.28 |
| *Buteo rufofuscus* |  |  |  |
| Medium fibres (rachis) | 50 | 234.26 | 35.15 |
